# Impact of intraoperative margin clearance on survival following pancreatoduodenectomy for pancreatic cancer: a systematic review and meta-analysis

**DOI:** 10.1038/s41598-020-79252-8

**Published:** 2020-12-17

**Authors:** Emrullah Birgin, Erik Rasbach, Patrick Téoule, Felix Rückert, Christoph Reissfelder, Nuh N. Rahbari

**Affiliations:** Department of Surgery, Universitätsmedizin Mannheim, Medical Faculty Mannheim, Heidelberg University, Theodor-Kutzer-Ufer 1-3, 68167 Mannheim, Germany

**Keywords:** Gastrointestinal cancer, Pancreatic cancer, Surgical oncology

## Abstract

The use of intraoperative margin revision to achieve margin clearance in patients undergoing pancreatoduodenectomy for pancreatic cancer is controversial. We performed a systematic review and meta-analysis to summarize the evidence of intraoperative margin revisions of the pancreatic neck and its impact on overall survival (OS). Nine studies with 4501 patients were included. Patient cohort was stratified in an R0R0-group (negative margin on frozen and permanent section), R1R0-group (revised positive margin on frozen section which turned negative on permanent section), and R1R1-group (positive margin on frozen and permanent section despite margin revision). OS was higher in the R1R0-group (HR 0.83, 95% CI 0.72–0.96, *P* = 0.01) compared to the R1R1-group but lower compared to the R0R0-group (HR 1.20; 95% CI 1.05–1.37, *P* = 0.008), respectively. Subgroup analyses on the use of different margin clearance definitions confirmed an OS benefit in the R1R0-group compared to the R1R1-group (HR 0.81; 95% CI 0.65–0.99, *P* = 0.04). In conclusion, intraoperative margin clearance of the pancreatic neck margin is associated with improved OS while residual tumor indicates aggressive tumor biology. Consensus definitions on margin terminologies, clearance, and surgical techniques are required.

## Introduction

Pancreatic cancer represents the seventh-leading cause of cancer deaths worldwide^1^. At present, treatment with curative intent encompasses surgical resection in combination with chemotherapy^2^. Despite recent advances in multi-disciplinary therapy, relapse rates are as high as 70% within 2 years^3^. The risk of tumor recurrence depends on the unique and aggressive biology of pancreatic cancer^4,5^. Pathological characteristics such as lymphovascular (LVI) and perineural invasion (Pn1), nodal metastases (N+), and positive resection margins (R+) were identified to be associated with a worse prognosis following resection^6^. The association of microscopic residual tumor at the resection margins with survival has remained a topic of controversial debates among clinicians^7,8^. In a more radical approach the aim of potentially curative surgery is to achieve clear margins, even if synchronous resections of adjacent organs or a complete pancreatectomy is required^9^. Therefore, at many centers intraoperative frozen section (FS) analyses of resection margins are routinely conducted and margin revision is performed in case of positive margins. An alternative approach considers margins to reflect the tumor biology with positive margins indicating an aggressive disease. In fact, the literature on the topic of intraoperative margin clearance and outcome following pancreatoduodenectomy (PD) for pancreatic cancer remains conflicting. Previous meta-analyses included up to five studies and therefore did not consider the entire currently available evidence^10–12^. The detailed location of positive margins on FS were not further outlined as well as the attempts of re-resection in case of positive FS. In addition, there were neither analyses regarding the applied definition of margin clearance between the resection groups, nor the rate of margin-clearance after resection in the previous reviews.

Therefore, the aim of the present systematic review and meta-analysis was to summarize the current evidence of intraoperative margin revisions of the pancreatic neck to achieve tumor clearance for patients undergoing PD for pancreatic cancer and its impact on survival.

## Methods

This systematic review and meta-analysis was reported in line with the Preferred Reporting Items for Systematic Reviews and Meta-Analysis (PRISMA) guidelines^13^. The study was performed according to guidance provided by the Cochrane Handbook for Systematic Reviews of Interventions^14^.

### Search strategy

A systematic database search of the MEDLINE, EMBASE, and Cochrane databases was done on March 27^th^, 2020 for all studies reporting on intraoperative FS evaluation during PD for pancreatic cancer. The detailed search strategy is provided as supplemental data. There were no language or time restrictions for the initial search. The retrieved title and abstract lists were independently scrutinized by two reviewers (EB, ER) to determine full-text eligibility. A third author (NNR) was consulted in case of disagreements between the two reviewers. Additional studies were added after a manual review of reference lists in relevant articles.

### Selection criteria

Studies were included if they reported about comparative data for intraoperative FS evaluation during PD for pancreatic ductal adenocarcinoma and corresponding survival data allowing for estimation of hazard ratios (HRs). All types of prospective and retrospective studies were included. Studies on patients (i) without data of intraoperative FS evaluation, (ii) without comparative data between the final margin status samples about long-term survival, (iii) with other pathologies than pancreatic cancer or no sufficient comparative data for pancreatic cancer in mixed cohorts, (v) with pancreatic resections other than PD (Whipple or pylorus-preserving pancreatic head resection), and (vi) in languages other than English, German, French or Spanish were excluded. Study protocols, review articles, letters, case reports or clinical series with a total sample size n < 10 were also excluded.

### Data extraction

The following data from eligible studies were extracted by two independent investigators (EB, ER): Author, publication date, study period, number of study centers, total number of patients, total number of FS analyses, patients’ age, gender of patients, neoadjuvant therapy, adjuvant therapy, pathological factors, postoperative morbidity, postoperative mortality, follow-up period, overall- and disease-free survival. For data extraction, the patient cohort was stratified as following: R0R0-resection (patients with a negative margin on FS and final histopathology), R1R0-resection (patients with a positive margin on FS, which turned negative on final histopathology after margin revision), R1R1-resection (patients with a positive margin on FS, which remained positive on final histopathology after margin revision). The evaluated margins included following overlapping margin terminologies: common bile duct/hepatic duct, pancreatic neck, peripheral pancreatic, uncinate process, superior mesenteric artery, superior mesenteric vein/portal vein groove, retroperitoneal, periuncinate retroperitoneal/mesopancreatic, circumferential anterior/posterior, posterior/inferior/superior pancreatic soft-tissue margin, enteral, and duodenum or stomach. Disagreements between the two reviewers (EB, ER) were resolved by a third author (NNR).

### Assessment of study quality

The Cochrane Collaboration’s ‘Risk of bias’ tool was used for assessing the methodological quality of the included studies^14^. The risk of bias domains was customized to the review question. The quality of the studies was assessed individually by two authors (EB, ER). Each of the following risk domains of bias was systematically categorized as ’high risk’, ’unclear risk’ and ’low risk’ of bias: selection bias, recall bias, attrition bias, analytical bias and reporting bias.

### Statistical analysis

The outcome measure was the hazard ratio (HR) for overall survival. In case of missing HRs and 95% confidence intervals (CIs) or standard errors (SEs), data was estimated according to the available survival data^15,16^. To perform meta-analyses, a minimum number of three studies was required. Heterogeneity between studies were explored by evaluating following factors, potentially, affecting overall survival: year of publication, study sample size, details of patients’ follow-up, details of histopathological assessments and outcome, applied margin terminology, details of intraoperative margin revision, reporting of false-negative rate and surgical strategy based on margin assessment. Subgroup analyses were performed if three or more studies revealed data for covariates affecting overall survival. Statistical analysis was performed using Review Manager Version 5.3 software (Cochrane Collaboration). The generic inverse-variance method was conducted using a random-effects model. Odds ratios (ORs) with 95% CI were calculated for binary outcomes. The interstudy heterogeneity (*I*^*2*^*)* was assessed using the *I*^*2*^value^17^. The significance level was set at *P* < 0.05. Publication bias was assessed using graphical funnel plot analyses^18^.

## Results

**Figure 1:**
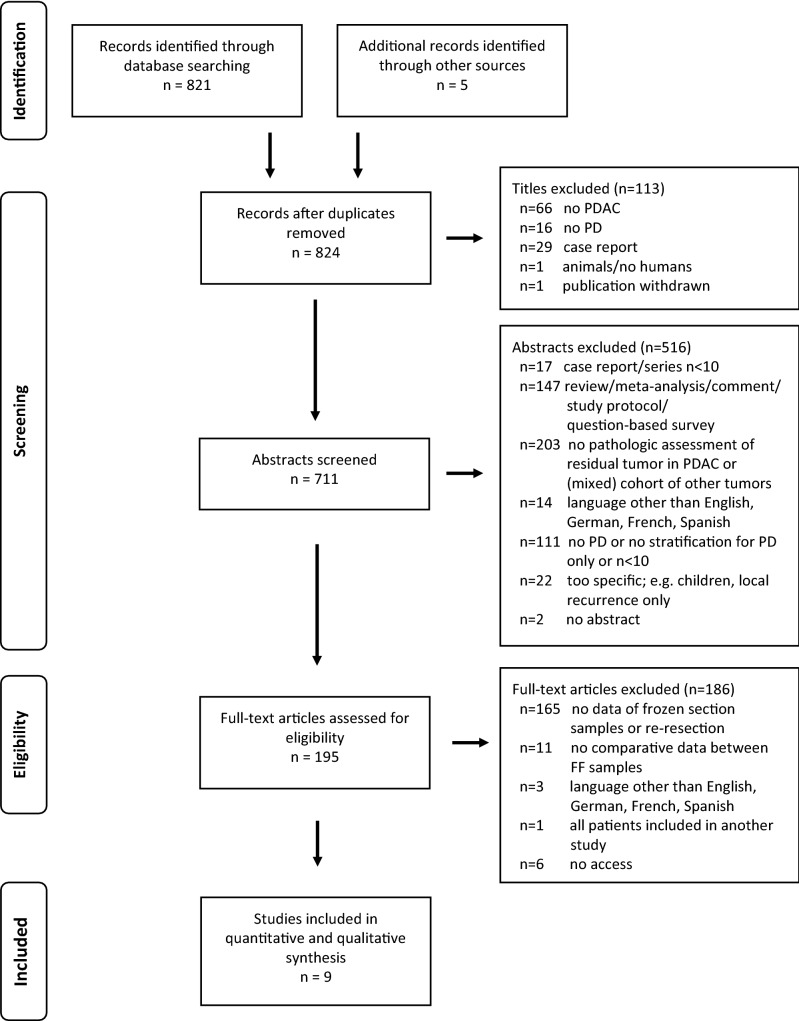
PRISMA flow chart.

A total of nine articles comprising 4501 patients were included in this meta-analysis^19–27^. Figure 1 displays the results of the search criteria according to the PRISMA guidelines^13^.

### Characteristics of included studies

The studies were published between 2009 and 2020 with a median study sample size of 448 patients. All included studies took place in referral centers for hepato-pancreato-biliary surgery in four different countries. The median follow-up was 19 months. Out of 4501 patients, a total of 1259 (28%) patients had positive tumor margins on final histopathological analysis. Overall, intraoperative FS analyses were performed in 4415 patients with comparative data, representing the final study cohort for meta-analysis. Table 1 outlines the main characteristics of the studies.

**Table 1:** Characteristics of included studies.

Author	Country	Year	Study	F/U	Patients			
			period	(mo)	Total	FSA (%)	Age	M:F
Crippa	Italy	2020	2010–2016	n/a	371^a^	371 (100)	70^b^	212:159
Fatima	US	2010	1981–2007	71	617	595 (96)	66	345:272
Hernandez	US	2009	1995–2009	17	202	202 (100)	66	105:97
Kooby	US	2014	2000–2012	20	1399	1399 (100)	65^c^	706:693
Mathur	US	2014	1995–2012	n/a	448	448 (100)	67	227:221
Nitschke	Germany	2017	1993–2014	16	301	262 (87)^d^	66	138:124
Pang	Australia	2014	2007–2012	16	116	101 (100)	68	64:52
Schmidt	US	2009	1992–2006	10–16^e^	61	51 (84)	64–69^f^	30:31
Zhang	US, Italy	2019	1998–2013	23	986	986 (100)	66^c^	503:483

*F/U* follow up (months), *FSA* frozen section analysis, *M:F* male-to-female ratio, *n/a* data not available.

^a^n = 182 were assessed for comparative frozen section analyses.

^b^n = 244 ≤ 70 years and n = 127 > 70 years.

^c^Values are presented as median.

^d^Secondary data analysis of individual dataset was performed, stratified by pancreatic ductal adenocarcinoma and pancreatoduodenectomy, patients with R2/Rx or no data for frozen section analyses were excluded as described in the original article.

^e^10 months F/U in the R1R1 group, 16 months F/U in the R1R0 group.

^f^Mean age 69 years in the R1R1 group, 64 years in R1R0 group.

Neoadjuvant therapy was administered in 489 of 3674 (13%) patients (Table S1)^19,20,22,24,26^. Of these, 108 patients (22%) had neoadjuvant chemotherapy, 34 (7%) received radiation therapy, and 347 (71%) underwent chemotherapy and/or radiation therapy, respectively. Adjuvant therapy was administered to 2915 out of 3937 (74%) patients^19,20,24–27^.

Five and four studies were single- and multi-center retrospective cohort studies, respectively. The quality assessment revealed a high risk of bias (Figure S1, Table S2). Assessment of the publication bias for the hazard ratio revealed a low asymmetry (Figure S2).

### Details of frozen section and permanent section analyses

A total of 3326 patients (74%) had tumor clearance on FS and final histopathological analysis (R0R0-group), 417 patients (9%) had residual tumor margins on FS which turned negative after margin revision on final histopathological analysis (R1R0-group), and 672 patients (15%) had residual tumor margins on final histopathological analysis despite intraoperative margin revision (R1R1-group). Some 100 margins (2%) with initially negative resection margins on FS had a positive resection margin on permanent section (false-negative findings) at the pancreatic neck (Table S3)^20,23,24,27^. On final histology, different margins were evaluated in all studies ranging between 3 and 8 margin locations.

### Details of margin revision

Positive resection margins on FS were detected in 1104 patients (25%). Of these, 887 patients (80%) had an intraoperative margin revision of the pancreatic neck (including other margins), but 420 resected margins (38%) remained positive. To achieve margin clearance, repetitive re-resections were performed in all studies. However, these were limited to the pancreatic neck^19–27^, bile duct^19,21,25^, and retroperitoneum^19^ including margins at the superior mesenteric artery and portal vein. Margin locations with persisting residual tumor following repetitive margin revision are detailed in Table S3 and stratified in “transection” and “dissection” margin. The attempt to revise the margin again after a second positive margin was described in four studies as “depending on the surgeon’s decision”^20–22,24^. Out of 1418 patients, 114 patients (8%) underwent total pancreatectomies due to repetitive positive margins at the pancreatic neck^24,26,27^. Resections of the portal vein or superior mesenteric vein, were performed in 845 out of 4440 patients (19%)^19–26^. Of these patients, 97 patients (11%) had positive resection margins on FS. One study showed aggressive extended resections due to residual tumors with resections of the hepatic artery and adjacent viscera^25^.

### Details of pathological assessment

The definition of residual tumor varied among included studies. Seven studies designated positive tumor margins as presence of cancer cells at the histological specimen^19–22,25–27^. Of these, two studies declared the deposits of high-grade dysplastic lesions in addition to cancer cells as positive resection margins^19,26^. Four studies^20,23,24,26^ defined residual tumor, if cancer cells were present within 1-mm of the resection margin and three studies^19,21,25^ if cancer cells were present at the resection edge. One study used both classifications depending on the recruitment period^22^. To classify the extent of pancreatic cancer, the 6th-8th editions of the American Joint Committee on Cancer Guidelines (AJCC) were used^19,20,24–26^. Two studies^23,26^ described the expertise of the pathologists involved in the frozen specimen assessment and two further studies^19,23^ gave details about the techniques of FS analyses.

### Meta-analysis of intraoperative margin revision and survival outcomes

**Figure 2:**
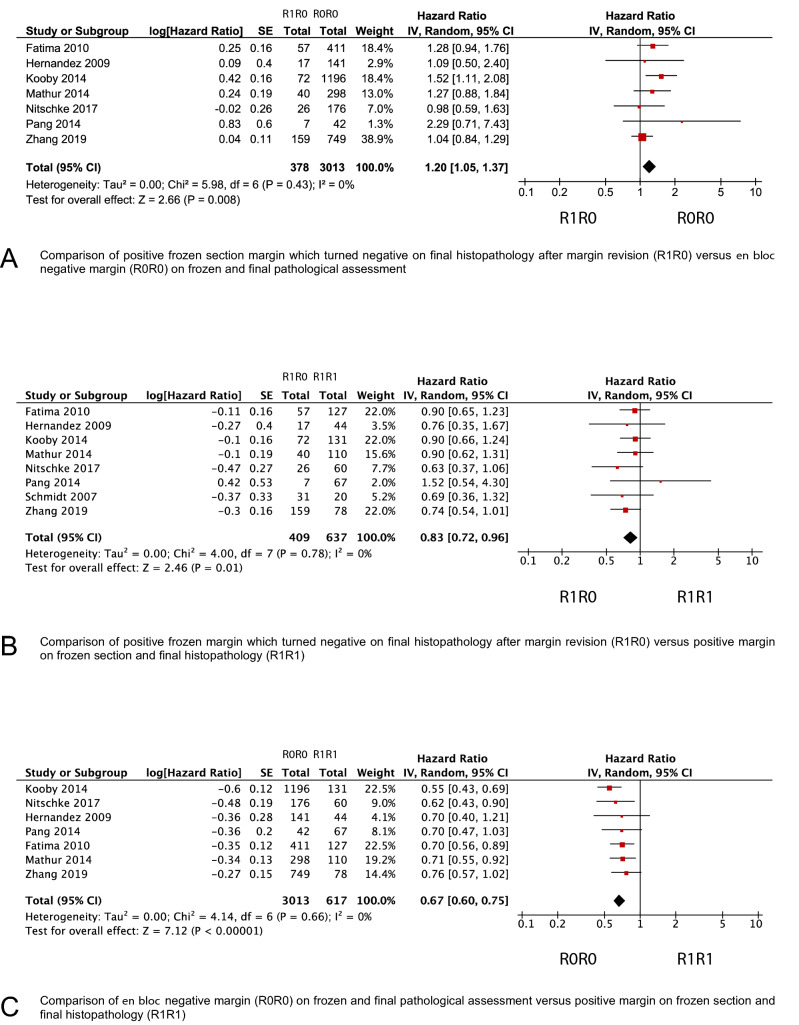
Forest plots comparing overall survival (**A**) in patients with secondary R0 resection after margin revision (R1R0-group) and *en bloc* R0-resection (R0R0-group), (**B**) in patients with R0 resection after margin revision (R1R0-group) and residual tumor on final assessment (R1R1-group), and in patients with *en bloc* R0-resection (R0R0-group) and residual tumor on final assessment (R1R1-group)*.* An inverse variance random effects model was used for meta-analysis. Squares and horizontal bars indicate point estimate (hazard ratios) with 95% CI for the individual studies.

**Figure 3:**
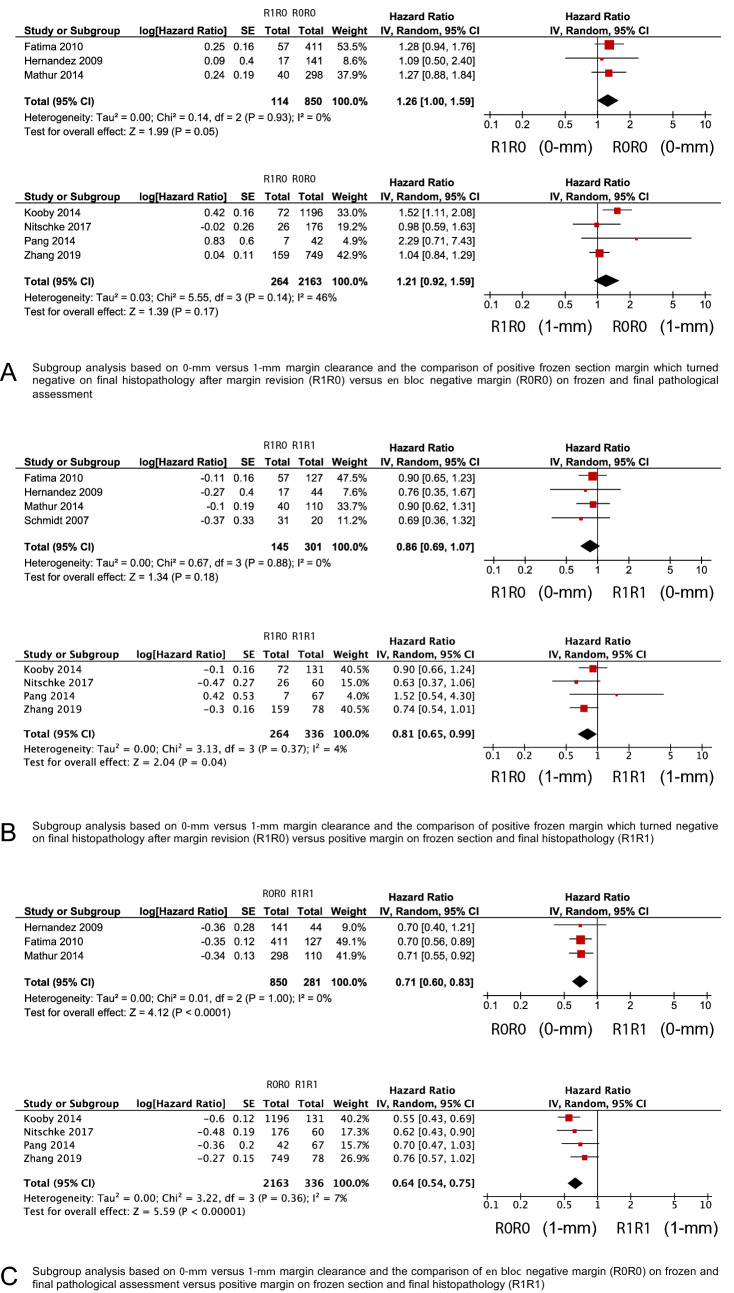
Subgroup analysis with forest plots comparing overall survival by using a 0-mm and 1-mm margin clearance (**A**) in patients with secondary R0 resection after margin revision (R1R0-group) and *en bloc* R0-resection (R0R0-group), (**B**) in patients with R0 resection after margin revision (R1R0-group) and residual tumor on final assessment (R1R1-group), and in patients with *en bloc* R0-resection (R0R0-group) and residual tumor on final assessment (R1R1-group)*.* An inverse variance random effects model was used for meta-analysis. Squares and horizontal bars indicate point estimate (hazard ratios) with 95% CI for the individual studies.

Median overall survival ranged between 18 and 29 months in the R0R0-group, 11–25 months in the R1R0-group, and 13 and 21 months in the R1R1-group, respectively (Table S4). Meta-analysis for overall survival revealed a significantly reduced risk of death in the R0R0-group compared to the R1R1-group (HR 0.67; 95% CI 0.60–0.75, *P* < 0.001) (Fig. 2). Patients in the R1R0-group showed increased survival compared to the R1R1-group (HR 0.83; 95% CI 0.72–0.96, *P* = 0.01) but decreased survival compared to the R0R0-group (HR 1.20; 95% CI 1.05–1.37, *P* = 0.008), respectively. Subgroup analyses for the use of the 1-mm margin clearance definition confirmed the survival benefit in the R1R0-group compared to the R1R1-group (HR 0.81; 95% CI 0.65–0.99, *P* = 0.04) but revealed no significant difference if 0-mm margin clearance was applied (HR 0.86; 95% CI 0.69–1.07, *P* = 0.18). In addition, patients in the R1R0-group had a comparable survival outcome compared to the R0R0-group in case of a 1-mm margin clearance (HR 1.21; 95% CI 0.92–1.59, *P* = 0.14) whereas 0-mm margin definitions were associated with a worse outcome (HR 1.26; 95% CI 1.00–1.59, *P* = 0.05). The subgroup of patients with R0R0-resection had a significantly better survival compared to the R1R1-group with 1-mm and 0-mm margin clearance, respectively (Fig. 3). Data on disease-free survival was reported in two studies^19,26^.

Subgroup analyses for the type of pathological assessments, aggressive treatment with repetitive pancreatic re-resections and extended resections, positivity of other resection margins and neoadjuvant/adjuvant treatment were not feasible due to missing stratified data for these covariates and the margin status as well as the high heterogeneity of reported data across the studies. These included further the use of different metrics (e.g. mean/median age vs. range), variable details of the surgical technique for PD or information regarding the level of lymphadenectomy, and missing stratifications for the study groups and subgroups which eventually precluded meta-regressions.

### Meta-analysis of intraoperative margin status as indicator of tumor biology

To test the hypothesis that intraoperative margins reflect patients’ tumor biology, we further assessed the outcomes of histopathological features between the study groups (Table S4). There was a trend for a lower odds-ratio of advanced tumor stage (T $\ge$ 3) in the R0R0-group compared to the R1R1-group (OR 0.67; 95% CI 0.43–1.04, *P* = 0.08), however, this did not reach statistical significance. The odds-ratio for nodal metastases in the R0R0-group was significantly lower compared to the R1R1-group (OR 0.61; 95% CI 0.41–0.91, *P* = 0.02) and higher in the R1R0-group compared to the R0R0-group (OR 1.89; 95% CI 1.00–3.59, *P* = 0.05), respectively. Further comparisons of advanced tumor stage, N+, poor histological grading (G $\ge$ 3), LVI, and Pn1 revealed no significant differences between the groups, respectively.

## Discussion

The present meta-analysis revealed that intraoperative margin clearance of the pancreatic neck was associated with improved overall survival for patients undergoing PD for pancreatic cancer. The survival benefit was even higher in patients with *en bloc* R0-resections as compared to both, intraoperative margin revisions and incomplete resections. However, we noticed several inconsistencies in the details of pathological assessments across the included studies reflecting the conflicting scientific landscape at its best with the ongoing debate on the correct definition of microscopic margin involvement after PD^28^. The AJCC defines in their current edition residual tumor as the presence of cancer cells within 1-mm of the margin. In contrast, the last edition of the International Union Against Cancer endorses a 0-mm clearance and considers the presence of tumor cells at the resection edge as a positive margin. Given the fact, that there is not even an international consensus on margin terminologies, none of the margins were reported consistently apart from the transection margins of the “pancreatic neck” and “common bile duct” (Table S3). In detail, Fatima et al. referred the retroperitoneal margin as a composite definition of the “uncinate process/SMA margin, and posterior, inferior, and superior soft tissue pancreatic margins “, whereas Mathur et al. described the retroperitoneal margin as the “SMA margin, and anterior and posterior sections of the uncinate margin”^19,21^. Kooby, Nitschke and Crippa et al. termed the retroperitoneal margin as the “SMA margin”^20,22,26^. Pang and Schmidt et al. applied the definition “periuncinate retroperitoneal margin” and “retroperitoneal/uncinate margins” as the retroperitoneal margin, respectively^23,27^. Hence, these variable designations of margins between the studies hamper definitive conclusions of the oncological impact of the retroperitoneal margin and represents a major source for heterogeneity. The complexity of the retroperitoneal region is further aggravated by the fact, that it is reported to be the most frequently positive margin after PD^6,29–31^. During PD, the tissue is dissected off the retroperitoneal space^32^. Therefore, FS analyses in this area are limited as are extensions of further resection unless concomitant arterial resections will be conducted^33^. The attempt to revise a margin varied among the studies. Despite aggressive re-resection approaches in the majority of studies, there was a clear disbalance of reported total pancreatectomies and extended resections^24,26,27^. The study by Crippa et al. concluded that conversion to total pancreatectomy is not associated with a survival benefit if compared to patients with PD (HR 1.89, 95% CI 1.08–3.31). Yet, more than 80% of their total pancreatectomy cohort had a residual tumor at the medial, retroperitoneal, and circumferential margins (1-mm). In contrast to this, the study by Schmidt et al. demonstrated a survival benefit if isolated intraoperative positive pancreatic neck margins (HR 0.69, 95% CI 0.36–1.32) were present (0-mm), but they excluded patients with R0R0-resections which further limits the validity of total pancreatectomy.

The implementation of standardized protocols for pathological workup rapidly increased the number of reported R1-rates from 20% to around 80%^34,35^. Moreover, rigorous pathological sectioning and sampling techniques were also associated with a higher rate of detecting R1-resections at permanent section analyses^9,36^. Although the pooled rate of false-negative results was below 2% across all studies, the study by Schmidt et al. reported a false-negative rate of 20% but outlined no further information of their pathological workup^27^. Diagnosis of malignant pancreatic lesions on FS can be challenging if chronic fibrosing pancreatitis is present^37^. The main drawback of its reliability is in case of a negative result as the sensitivity ranges between 33 and 80% while having a specificity of 100%^38^. As the use of neoadjuvant therapy for borderline resectable pancreatic cancer is further emerging, the accuracy of FS analyses might be restricted^39^. Nevertheless, the false-negativity rate of FS analyses was 3% and 4% in the studies including neoadjuvant therapies^20,24^ but the pathological assessment of FS was briefly mentioned in two studies^19,23^.

Recent studies determined the involvement of multiple margins and individual margins to be an independent prognostic factor^40,41^. However, positive resection margins might also indicate a biologically more aggressive tumor rather than simply reflecting a meticulous pathological sampling or insufficient surgical technique^42^. Recurrent disease after surgery is frequently observed, so that pancreatic cancer remains a systemic disease^43,44^. Although isolated local recurrence is less frequent than distant metastasis, the long-term survival is similar^45^. We identified distinct pathological characteristics between the study groups suggesting an advanced disease in the R1R1-group and R1R0-group as nodal metastases were more present in these groups compared to the R0R0-group. Moreover, 50% of the margin revisions in the studies failed to achieve negative margins, implying this theory of more advanced tumor biology in the R1R0- and R1R1-group. Notwithstanding that other prognostic histopathological features as lymphovascular and perineural invasion were not significantly different, one has to consider that less than half of the included studies disclosed these pathological characteristics in their analyses which were responsible for heterogeneity between the studies.

There are several limitations to this meta-analysis. All included studies were retrospective studies with a considerable selection bias. The simultaneous positivity of other margins was not amenable to further subgroup analyses or meta-regressions as were other important covariates such as specific neoadjuvant or adjuvant approaches. Although we detected several factors causing heterogeneity in the present study, meta-regressions were not feasible and a random-effects model was assumed as unexplained heterogeneity might still remain between the included studies (e.g. different sampling techniques). Certainly, the main bias in the studies was the use of heterogeneous definitions of margin clearance as well as margin terminologies although recent studies adopted the new R-status of 1-mm clearance^46,47^. The overall survival benefit of intraoperative margin revision was confirmed by a subgroup analysis of studies applying the 1-mm clearance. This might explain why we observed a survival benefit in our analysis in contrast to the previously published meta-analyses despite the range of reported R1-rates were identical. Still, the effort of intraoperative tumor clearance at the pancreatic neck should respect the tumor biology. Therefore, we recommend at our institution a revision of the pancreatic neck only if none of the other margins remain positive. Ideally, the benefit of intraoperative margin clearance should be addressed in a randomized trial comparing FS analyses to achieve margin clearance in borderline resectable pancreatic cancer with standardized pathological protocols, surgical techniques as well as clearly defined margins. In conclusion, precise surgical techniques combined with standardized pathological assessments are currently the major pillars of a survival benefit in the backdrop of neoadjuvant and immunooncologic approaches following PD^48^.

## Supplementary Information

Supplementary Information 1.

## Data Availability

The datasets generated during and/or analysed during the current study are available from the corresponding author on reasonable request.
