# Supplementary material for: Impact of intraoperative margin clearance on survival following pancreatoduodenectomy for pancreatic cancer: a systematic review and meta-analysis
Source: Sci Rep. 2020 Dec 17;10:22178. doi: 10.1038/s41598-020-79252-8 (PMC7746710; doi:10.1038/s41598-020-79252-8)
Supplement: Supplementary file 1 — Supplementary Information 1. [file 41598_2020_79252_MOESM1_ESM.docx]

**Impact of Intraoperative Margin Clearance on Survival Following Pancreatoduodenectomy for Pancreatic Cancer: A Systematic Review and Meta-Analysis**

Emrullah Birgin (EB)^1^*, Erik Rasbach (ER)^1^*, Patrick Téoule (PT)^1^, Felix Rückert (FR)^1^, Christoph Reissfelder (CR)^1^, Nuh N. Rahbari (NNR)^1#^

^1^ Department of Surgery, Universitätsmedizin Mannheim, Medical Faculty Mannheim, Heidelberg University, Mannheim, Germany

**SUPPLEMENTAL DATA**

**Search Method**

**Table S1:** Overview of neoadjuvant and adjuvant treatment modalities

**Table S2:** Characteristics of risk of bias assessment

**Table S3:** Characteristics of resection margins and intraoperative margin revision

**Table S4**: Summary of survival outcome and histopathological characteristics

**Figure S1:** Risk of bias assessment

The risk domains of bias (selection bias, recall bias, attrition bias, analytical bias and reporting bias) was categorized by ’high risk’, ’unclear risk’ and ’low risk’ of bias

**Figure S2:** Publication bias assessment of the hazard ratios *(A)* in patients with secondary R0 resection after margin revision (R1R0-group) vs. *en bloc* R0-resection (R0R0-group), *(B)* in patients with R0 resection after margin revision (R1R0-group) vs. residual tumor on final assessment (R1R1-group), and in patients with *en bloc* R0-resection (R0R0-group) vs. residual tumor on final assessment (R1R1-group)*.*

**Search method**

("pancreatic cancer"[All Fields] OR "pancreatic carcinoma"[All Fields] OR "PDAC"[All Fields]) AND ("pancreaticoduodenectomy"[All Fields] OR "pancreatic head resection"[All Fields] OR "pancreatoduodenectomy"[All Fields] OR "Whipple"[All Fields]) AND ("resection margin"[All Fields] OR "pancreatic neck margin"[All Fields] OR "intraoperative" OR "frozen section"[All Fields] OR “frozen"[All Fields] OR "margin"[All Fields] OR "re-resection"[All Fields] OR “r-status”[All Fields] OR “pathology” [All Fields]) AND (“survival” [All Fields])

**Search date: 03/27/2020**

**Table S1:** Neoadjuvant and adjuvant treatment

| **Author** |  | **Neoadjuvant CTx/RTx (%)** | | | |  | | **Adjuvant CTx/RTx (%)** | | |
| --- | --- | --- | --- | --- | --- | --- | --- | --- | --- | --- |
|  |  | **Total** | **R0R0** | **R1R0** | **R1R1** |  | **Total** | **R0R0** | **R1R0** | **R1R1** |
| Crippa |  | 96 (26) | n/a | n/a | n/a |  | 315 (85) | n/a | n/a | n/a |
| Fatima ^a^ |  | 46 (7) | n/a | n/a | n/a |  | 448 (73) | 327 (53) ^b^ |  | 121 (20) |
| Hernandez |  | n/a | n/a | n/a | n/a |  | 188 (93) | 127 (63) | 17 (8) | 44 (22) |
| Kooby |  | 136 (10) | 118 (8) | 7 (1) | 11 |  | 960 (69) | 823 (59) | 50 (4) | 87 (6) |
| Mathur |  | n/a | n/a | n/a | n/a |  | n/a | n/a | n/a | n/a |
| Nitschke |  | 20 (4) | 15 (3) | 1 (1) | 4 |  | 164 (54) | 107 (36) | 17 (6) | 40 (13) |
| Pang |  | n/a | n/a | n/a | n/a |  | n/a | n/a | n/a | n/a |
| Schmidt |  | n/a | n/a | n/a | n/a |  | 29 (48) | n/a | 16 (26) | 13 (21) |
| Zhang |  | 191 (19) | 138 (14) | 34 (3) | 19 |  | 811 (82) | 622 (63) | 127 (13) | 62 (6) |
| **Total** |  | **489/3674 (13)** | **271/2686 (10)** | **42/2686 (2)** | **34/2686 (1)** |  | **2915/3937 (74)** | **2006/3505 (57)** | **227/2949 (8)** | **367/3566 (10)** |

^a^ n=22 patients had R2 resections

^b^ data only available for R0R0 and R1R0 resections

R0R0 negative margin, R1R0 secondary negative margin after intraoperative revision, R1R1 residual tumor; CTx chemotherapy, RTx radiation therapy,n/a data not available

**Table S2:** Characteristics of risk of bias assessment

| **Author** | **Selection bias** | **Recall bias** | **Attrition bias** | **Analytical bias** | **Reporting bias** |
| --- | --- | --- | --- | --- | --- |
|  |  |  |  |  |  |
| **Fatima** | Inclusion of consecutive patients over a 26-year recruitment period with only confirmed pathological results | Retrospective study | Significant missing parameters (e.g. stratification for TNM, number of positive frozen section margins)  No definition of residual tumor | Effect of *en-bloc* R0-resection (R0R0-group) and the implications of positive resection margins on survival, however, positivity of other margins was accepted (e.g. superior mesenteric artery) and not described in the aim of the study | Missing data of significant parameters, e.g. postoperative complications and histologic grading |
|  |  |  |  |  |  |
| **Kooby** | Inclusion of consecutive patients across 8 medical centers | Retrospective study | Mismatch of data between the groups | Impact of margin clearance at the pancreatic neck on survival, but the positivity of other margins was accepted (e.g. superior mesenteric artery) and not described in the aim of the study | Missing data of significant parameters, e.g. postoperative complications |
|  |  |  |  |  |  |
| **Mathur** | Inclusion of consecutive patients at a single center | Retrospective study | Missing detailed follow-up data  No definition of residual tumor | Impact of different pancreatic neck margin status on survival (other resection margins were negative on final histology) | Missing data of significant parameters, e.g. postoperative complications and repetitive revised margins |
|  |  |  |  |  |  |
| **Nitschke** | Inclusion of consecutive patients at a single center | Retrospective study | No stratification of malignancies and operative techniques, however, primary data was available  No definition of residual tumor | Impact of different pancreatic neck margin status on survival (other resection margins were negative on final histology) | Missing data of significant parameters, e.g. postoperative complications and repetitive revised margins |
|  |  |  |  |  |  |
| **Pang** | Inclusion of consecutive patients across 4 medical centers | Retrospective study | Missing detailed follow-up data | Impact of different margin status on survival, however, irrespective of other margins | Missing data of significant parameters, e.g. postoperative complications and repetitive revised margins |
|  |  |  |  |  |  |
| **Zhang** | Different study periods between two study centers (Historical bias) | Retrospective study | No definition of residual tumor | Impact of different margin status on survival, however, irrespective of other margins | Detailed information on relevant parameters, however, only pancreatic neck margin assessed for long-term survival |
|  |  |  |  |  |  |
| **Hernandez** | Inclusion of consecutive patients at a single center | Retrospective study | No definition of residual tumor  Complete follow-up data | Impact of different margin status on survival and extended pancreatic resections, however, irrespective of other margins | Missing data of significant parameters, e.g. postoperative complications, pathological characteristics and repetitive revised margins |
|  |  |  |  |  |  |
| **Crippa** | Inclusion of consecutive patients across 3 medical centers in the same recruitment period | Retrospective study | Missing follow-up data and stratification of patients according to the resection margins | Impact of different margin status on survival and tumor recurrence, however, irrespective of other margins and missing data for overall survival | Missing data of significant parameters, e.g. postoperative complications, pathological characteristics and repetitive revised margins |
|  |  |  |  |  |  |
| **Schmidt** | Inclusion of consecutive patients in two centers | Retrospective study | Exclusion of *en-bloc* resected cancer  No definition of residual tumor | Impact of total pancreatectomy and margin positivity at the pancreatic neck on survival (other margins were not described) | 10 out of 61 patients had no frozen section analyses and missing data of significant parameters, e.g. postoperative complications |

Selection bias indicates bias due to non-inclusion of consecutive patients; recall bias, bias due to retrospective data collection; attrition bias, bias due to reporting of incomplete data (e.g. loss to follow up, mortality, perioperative complications); analytical bias, bias due to mismatch of aim of study and outcome evaluation; reporting bias, bias due to limited availability of data/selective reporting of data.

**Table S3:** Characteristics of resection margins and intraoperative margin revision

| **Author** | **Margin revision (%)** | | |  |  | **Transection margin^a^ (%)** | | |  | **Dissection margin^b^ (%)** | | |
| --- | --- | --- | --- | --- | --- | --- | --- | --- | --- | --- | --- | --- |
| **(FSA)** | **R+ on frozen section** | **Attempts of re-resection** | **Persistent frozen R+ margin** | **R+ on permanent section** |  | **Neck** | **CBD** | **Enteric** |  |  | | |
| Crippa  (n=371) | 58 (16) | 58 (16) | 0 (0) | 239 (64) | R1R0  R1R1^c^ | 58 (16)  0 (0) | 0 (0)  0 (0) | 0 (0)  0 (0) |  | ø  239 (64)  *uncinated/retroperitoneal (incl. SMA / vein margins), circumferential anterior, posterior, and medial margins* | | |
| Fatima  (n=595) | 184 (31) | 184 (30) | 127 (21) | 149 (24) | R1R0  R1R1^c^ | 23 (4)  32 (5) | 3 (0)  4 (1) | 0 (0)  0 (0) |  | 18 (3)  110 (18)  *uncinate (SMA) margin* | 0 (0)  13 (2) *peripancreatic soft tissue* | 6 (1)  50 (8)  *PV* |
| Hernandez  (n=202) | 61 (30) | 61 (30) | 44 (22) | 44 (22) | R1R0  R1R1^c^ | 16 (8)  21 (10) | 2 (1)  16 (8) | ø  ø |  | ø  12 (6) *uncinate margin/ SMA* | ø  17 (8)  *peripheral pancreatic margin* | ø  5 (2)  *PV* |
| Kooby  (n=1399) | 203 (15)  (FN: n=51) | 118 (8) | 46 (3) | 351 (25) | R1R0  R1R1^c^ | 72 (5)  131 (9) | ø  n/a | ø  ø |  | ø  264 (19) *SMA margin* |  |  |
|  |  |  |  |  |  |  |  |  |  |  |  |  |
| Mathur  (n=448) | 150 (33) | 150 (33) | 110 (25) | 110 (25) | R1R0  R1R1 | n/a  n/a | n/a  n/a | n/a  n/a |  | n/a  n/a  *SMA, anterior, posterior, uncinate and ampullary margin* | | |
| Nitschke  (n=262) | 86 (29) | 86 (29) | 60 (20) | 60 (20) | R1R0  R1R1 | n/a  n/a | n/a  n/a | ø  ø |  | n/a  n/a *retroperitoneal margin (posterior and SMA)* | | |
| Pang  (n=101) | 74 (58)  (FN: n=2) | 19 (16) | 12 (10) | 67 (58) | R1R0  R1R1^c^ | 7 (6)  7 (6) | ø  0 (0) | ø  0 (0) |  | ø  40 (34) *periuncinate retroperitoneal soft tissue margin* | ø  25 (22)  *anterior surface of the pancreas and true posterior margin* | ø  42 (36) *SMV bed* |
| Schmidt  (n=51) | 51 (100)  (FN: n=11) | 31 (51) | 0 (0) | 28 (46) | R1R0  R1R1 | 31 (51)  28 (46) | ø  0 (0) | ø  ø |  | ø  0 (0)  *retroperitoneal margin* | | |
| Zhang  (n=986) | 237 (24)  (FN: n=36) | 180 (18) | 21 (2) | 211 (21) | R1R0  R1R1^c^ | 159 (16)  78 (8) | ø  n/a | ø  n/a |  | ø  147 (15) *SMA/ PV margin* | ø  n/a *posterior margin* |  |
| **Total**  **(n=4415)** | **1104**  **(25%)**  **(FN: n=100)** | **887**  **(20%)** | **420**  **(9%)** | **1259**  **(28%)** | **R1R0**  **R1R1** | **366/3752**  **297/3752** | **5/1190**  **20/1367** | **0/988**  **0/1104** |  |  |  |  |

FSA total number of frozen section analyses excluding R2 resections, R+ residual tumor, R1R0 margin cleared after initially positive frozen section, R1R1 margin involvement on permanent section analysis, CBD common bile duct, SMA superior mesenteric artery, PV portal vein, SMV superior mesenteric vein, n/a quantitative data not available , ø assessment and/or re-resection not performed, FN false-negative analyses

^a^ values represent the number of margins assessed and/or revised

^b^ secondary data analysis of individual dataset was performed, stratified by pancreatic ductal adenocarcinoma and pancreaticoduodenectomy, patients with R2/Rx or no data for frozen section analyses were excluded as described in the original article

^c^ values include residual tumor without prior frozen section analysis

**Table S4:** Summary of survival outcome and histopathological characteristics

|  |  |  | | | |  | **Odds Ratio (95% CI)** |  |
| --- | --- | --- | --- | --- | --- | --- | --- | --- |
| **Author** | **Variable** | **Total** | **R0R0** | **R1R0** | **R1R1** | **R0R0 vs. R1R1** | **R1R0 vs. R1R1** | **R1R0 vs. R0R0** |
| Crippa | Total (n) Median DFS (mo) | 371 - | 313 20 | 58 12 | 0 - | - | - | - |
| Fatima | Total (n)  Median OS (mo)  Median DFS (mo) | 617 ^a^ 18 ^b^ - | 411  19 14 | 57  18 12 | 127  15 12 | -  - | -  - | -  - |
|  |  |  |  |  |  |  |  |  |
| Hernandez | Total (n)  Median OS (mo)  $\geq$ T3  N^+^ | 202 17 123/202 115/202 | 141  21 83/141 73/141 | 17  11 12/17 15/17 | 44  13 28/44 27/44 | -  - 0.82 (0.41, 1.65) 0.68 (0.34, 1.35) | -  - 1.37 (0.41, 4.60) 4.72 (0.96, 23.28) | -  - 1.68 (0.56, 5.02) 6.99 (1.54, 31.69) |
| Kooby | Total (n)  Median OS (mo)  $\geq$ T3  N^+^  Pn1 LVI  $\geq$ G3 | 1399 20 1101/1388 949/1399 1044/1399 625/1399 442/1370 | 1196 21 938/1185 797/1196 873/1196 531/1196 382/1170 | 72  12 58/72 55/72 58/72 30/72 25/72 | 131 14 ^c^ 105/131 97/131 113/131 64/131 35/128 | -  - 0.94 (0.60, 1.48) 0.70 (0.47, 1.05) 0.43 (0.26, 0.72) 1.12 (0.70, 1.81) 0.86 (0.53, 1.40) | -  - 1.03 (0.50, 2.12) 1.13 (0.58, 2.22) 0.66 (0.31, 1.42) 0.75 (0.42, 1.34) 0.95 (0.48, 1.86) | -  - 1.09 (0.60, 1.99) 1.62 (0.93, 2.83) 1.53 (0.84, 2.79) 0.89 (0.55, 1.45) 1.10 (0.67, 1.81) |
| Mathur | Total (n)  Median OS (mo)  $\geq$ T3  N^+^ | 448 n/a 328/448 274/448 | 298 20 201/298 169/298 | 40 14 33/40 31/40 | 110 12 93/110 74/110 | -  - 0.38 (0.21, 0.67) 0.64 (0.40, 1.01) | -  - 0.86 (0.33, 2.26) 1.68 (0.72, 3.89) | -  - 2.28 (0.97, 5.33) 2.63 (1.21, 5.72) |
| Nitschke ^e^ | Total (n)  Median OS (mo)  $\geq$ T3  N^+^  Pn1 LVI  $\geq$ G3 | 301 ^d^ n/a 239/262 174/262 191/261 145/261 113/248 | 176 24 161/176 101/176 131/175 88/175 71/164 | 26 25 24/26 21/26 17/26 14/26 14/26 | 60 13 54/60 52/60 43/60 43/60 28/58 | -  - 1.19 (0.44, 3.23) 0.21 (0.09, 0.46) 1.18 (0.61, 2.27) 0.39 (0.21, 0.74) 0.82 (0.46, 1.49) | -  - 1.33 (0.25, 7.09) 0.65 (0.19, 2.20) 0.75 (0.28, 2.00) 0.46 (0.18, 1.20) 1.25 (0.49, 3.16) | -  - 1.12 (0.24, 5.20) 3.12 (1.12, 8.65) 0.63 (0.26, 1.53) 1.15 (0.50, 2.63) 1.53 (0.67, 3.51) |
| Pang | Total (n)  Median OS (mo)  $\geq$ T3  N^+^  Pn1  $\geq$ G3 | 116 25 107/116 86/116 83/116 36/115 | 42 29 35/42 27/42 22/42 14/41 | 7 16 6/7 5/7 4/7 2/7 | 67 ^f^ 23 ^f^ 66/67 54/67 57/67 20/67 | -  - 0.08 (0.01, 0.64) 0.43 (0.18, 1.04) 0.19 (0.08, 0.48) 1.22 (0.53, 2.80) | -  - 0.09 (0.01, 1.64) 0.60 (0.10, 3.46) 0.23 (0.05, 1.21) 0.94 (0.17, 5.26) | -  - 1.20 (0.12, 11.58) 1.39 (0.24, 8.05) 1.21 (0.24, 6.09) 0.77 (0.13, 4.49) |
| Schmidt | Total (n)  Median OS (mo)  N^+^ | 61 ^g^ n/a 41/63 | n/a n/a n/a | 31 18 21/33 | 20 10 20/20 | -  - - | -  - 0.04 (0.00-0.76) | -  - |
|  |  |  |  |  |  |  |  |  |
| Zhang | Total (n)  Median Survival (mo)  $\geq$ T3  N^+^  Pn1  LVI  $\geq$ G3 | 986 26 145/986 781/986 913/986 738/986 315/941 | 749 28 108/749  601/749 691/749 557/749 236/714 | 159 24 22/159  120/159 150/159 127/159 52/152 | 78 19 15/78  60/78 72/78 54/78 35/75 | -  - 0.71 (0.39, 1.29)  1.22 (0.77, 2.13) 0.99 (0.41, 2.38) 0.58 (0.34, 0.99) 0.61 (0.38, 0.97) | -  - 0.67 (0.33, 1.39)  0.92 (0.49, 1.75) 1.39 (0.48, 4.05) 1.76 (0.95, 3.27) 0.92 (0.52, 1.63) | -  - 0.71 (0.39, 1.29)  0.76 (0.51, 1.13) 1.40 (0.68, 2.89) 1.37 (0.90, 2.08) 1.05 (0.73, 1.52) |
| **Total** | Total (n) Median OS (mo) $\geq$ T3 N^+^ Pn1 LVI $\geq$ G3 | **4501  2042/3402 2379/3413 2231/2762 1508/2646 914/2674** | **3326**  **19-29**  **1526/2591**  **1768/2602 1717/2162 1176/2120 703/2089** | **467**  **11-25**  **155/321**  **247/321 229/264 171/257 93/257** | **637**  **10-23**  **361/490**  **364/490 285/336 161/269 118/328** | **0.67 (0.43, 1.04); P=0.08**  **0.61 (0.41, 0.91); P=0.02 0.57 (0.27, 1.19); P=0.13 0.65 (0.35, 1.20); P=0.17 0.92 (0.62, 1.37); P=0.70** | **0.87 (0.58, 1.31); P=0.51**  **1.05 (0.61, 1.81); P=0.85 0.73 (0.45, 1.18); P=0.20 0.90 (0.43, 1.87); P=0.77 0.83 (0.64, 1.34); P=0.69** | **1.19 (0.86, 1.64); P=0.29**  **1.89 (1.00, 3.59); P=0.05 1.19 (0.86, 1.64); P=0.31 1.14 (0.86, 1.64); P=0.38 1.10 (0.84, 1.45); P=0.49** |

R0R0 negative margin on frozen and permanent section, R1R0 secondary R0 resection after margin revision, R1R1 residual tumor on frozen and permanent section; DFS disease-free survival; OS overall survival
^a^ n=22 patients had R2 resections

^b^ median survival in the R2 resection group: 10 months

^c^ includes false negative frozen section analyses

^d^ n=39 patients had no frozen section analyses

^e^ secondary data analysis of individual dataset was performed, stratified by pancreatic ductal adenocarcinoma and pancreaticoduodenectomy, patients with R2/Rx or no data for frozen section analyses were excluded as described in the original article

^f^ n=15 patients had no frozen section analyses

^g^ n=10 patients had no frozen section analysis

n/a data not available

**Figure S1:** Risk of bias assessment


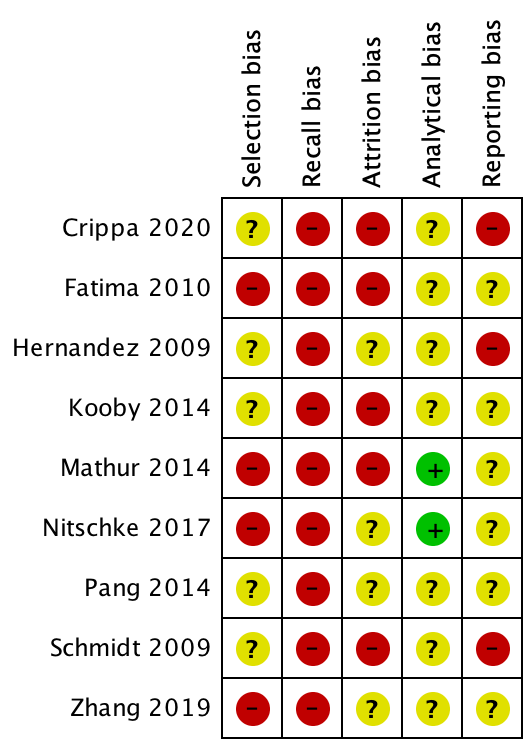


**Figure S2:** Publication bias assessment
